# Editorial: Congenital and perinatal infections: How to prevent sequelaes in neonates and children

**DOI:** 10.3389/fped.2023.1142636

**Published:** 2023-02-13

**Authors:** Domenico Umberto De Rose, Maria Paola Ronchetti, Chryssoula Tzialla, Mario Giuffré, Cinzia Auriti

**Affiliations:** ^1^ Neonatal Intensive Care Unit, “Bambino Gesù” Children’s Hospital IRCCS, Rome, Italy; ^2^ Neonatal and Pediatric Unit, Polo Ospedaliero Oltrepò, ASST Pavia, Pavia, Italy; ^3^ Department of Health Promotion, Mother and Child Care, Internal Medicine and Medical Specialties “G. D’Alessandro”, University of Palermo, Palermo, Italy

**Keywords:** cytomegalovirus, toxoplasma, congenital syphilis, neonatal sepsis, COVID-19

**Editorial on the Research Topic** Congenital and perinatal infections: How to prevent sequelaes in neonates and children

The current severe acute respiratory syndrome coronavirus-2 (SARS-CoV-2) pandemic has overwhelmingly absorbed attention and health resources for 2 years, allowing us to reflect that infections are a permanent health and social problem, causing morbidity and mortality. They require organization, important prevention measures, and containment. This is particularly true in the neonatal age, where infections remain a complex problem with serious consequences.

The reduction in under-5 mortality by more than 50% observed in the last 25 years shows us that healthcare has achieved unprecedented goals and successes. However, neonates represent a different story: neonatal mortality decreased much more slowly in the same period (1). Therefore, reducing neonatal mortality should be at the heart of international policies to contrast this trend. Considering that infections are still one of the leading causes of neonatal death worldwide after prematurity and perinatal asphyxia, we cannot eliminate neonatal mortality without eliminating avoidable infections. Most of these cases are sepsis, and the “get to zero infections” must not be a goal of high-income countries alone. Beyond mortality, neonatal sepsis is still burdened by a high rate of neurodevelopmental disability (2). Further studies are needed to explore the neurodevelopmental outcomes according to the different sepsis risk assessment and management approaches in term and preterm infants. The changing epidemiology of involved bacteria and the new antibiotic resistance data should also be kept in mind to improve outcomes (3).

Some infections are contracted from the mother during pregnancy and transmitted to the fetus (congenital infections), during labor and childbirth (perinatal infections), and throughout breastfeeding (postnatal infections). The microorganisms most frequently responsible for these categories of infections are not just bacteria: Cytomegalovirus, Toxoplasma gondii, Treponema pallidum, Hepatitis B and C viruses, Human Immunodeficiency Virus, Parvovirus B19, Rubella, and non-polio Enterovirus. Currently, these infections are known under the acronym TORCH (T for Toxoplasmosis, O for other Agents, R for Rubella, C for Cytomegalovirus, and H for Herpes viruses). The SARS-CoV-2 and Zika virus, until now little or known at all, have attracted attention, the first due to the pandemic diffusion and the unknown transmissibility to the fetus when contracted during pregnancy; the second for the teratogenic potential, which appears increasingly clear. These infections in pregnancy may lead to spontaneous abortion, fetal death, or intrauterine growth retardation or can cause congenital anomalies and sequelae of different severity (4).

Specific risk factors may influence the incidence of the transmission to the fetus and the severity of sequelae: timing of infection in pregnancy, order of the infection (primary or reinfection or chronic one), duration of maternal rupture of membranes, route of delivery, socio-economic conditions, and breastfeeding. Many of the harmful effects of these infections on the newborn could be reduced or sometimes eliminated if screening and prevention activities are practiced in a timely manner and if access to prevention services is simple, even for more disadvantaged women.

Prenatal screening programs for congenital infections can help avoid mother-to-fetus transmission and advise appropriate and prompt treatment and/or counseling to prevent major sequelae in neonates and children. To date, fetal infections are avoidable, totally or in part, by specific drugs, vaccines, or passive immunization administered to pregnant women; many diagnostic tests can help doctors with appropriate prenatal counseling, guiding families with therapies and decisions. For example, a recent meta-analysis confirmed that a negative amniocentesis in pregnant women with Cytomegalovirus (CMV) infection ensures the lack of fetal insult and long-term sequelae to the child, even if the transmission has occurred (5).

Concerning congenital and perinatal infections, neonates with overt symptoms at delivery have a poorer prognosis than asymptomatic ones; however, long-term sequelae (mental and sensorineural sequelae) may occur also in infected children, which are asymptomatic at birth, after the first year of life (6). Most ophthalmological and auditory anomalies might be also progressive. New viruses also, such as Zika and SARS-CoV-2, are confirmed to be responsible for potential disabilities, with motor abnormalities and epilepsy in infants and children with evidence of congenital Zika Virus infection (7), and individual developmental disorders and abnormal ophthalmological findings after exposure to maternal SARS-CoV-2 infection in pregnancy (8, 9). Furthermore, viral infections may also cause autism spectrum disorders (ASDs) through direct teratogenic effects and indirect impacts on the developing brain from inflammation or maternal immune activation. Long-term monitoring is mandatory for children whose mothers report an inflammatory episode of viral infection at any time during pregnancy (10). In the absence of a long-term follow-up, sequelae in neonates and children are frequently unanticipated with a possible poor prognosis.

In the case of mothers with a history of certain or suspected infections in pregnancy, a timely screening of their neonates in the first weeks after birth can be crucial to diagnose congenital infections and to determine the infection course, as in congenital CMV infections (11). Because of the rapid method for detecting the DNA - CMV in saliva and the efficacy of the antiviral therapy in symptomatic infants, CMV screening is cost-effective (12). Interestingly, many cases without prenatal/neonatal signs of congenital CMV infection or maternal history of CMV infection can be identified only by universal screening (13). Without it some infected children who can develop late neurological sequelae may go unnoticed, depriving them of early access to instrumental and therapeutic measures. Therefore, further studies are needed to guide public health institutions to improve the outcomes of tomorrow’s adults.
